# Retraction Note: Exosomes from glioma cells induce a tumor-like phenotype in mesenchymal stem cells by activating glycolysis

**DOI:** 10.1186/s13287-021-02299-5

**Published:** 2021-04-14

**Authors:** Zhanjun Ma, Xue Cui, Li Lu, Guohu Chen, Yang Yang, Yan Hu, Yubao Lu, Zhangqi Cao, Yan Wang, Xuexi Wang

**Affiliations:** 1 The Second Clinical Medical College, Lanzhou University, Lanzhou, 730000 Gansu China; 2 The First Clinical Medical College, Lanzhou University, Lanzhou, 730000 Gansu China; 3 Institute of Pharmacology, School of Basic Medical Science, Lanzhou University, Lanzhou, 730000 Gansu China; 4 Key Laboratory of Preclinical Study for New Drugs of Gansu Province, Lanzhou, 730000 Gansu China; 5 School of Basic Medical Sciences of Lanzhou University, School of Medicine, 205 Tianshui Rd South, Lanzhou, 730000 Gansu China; 6 School of Basic Medical Sciences, Lanzhou University, Lanzhou, 730000 Gansu China; 7 School of Basic Medical Sciences, Lanzhou University, Lanzhou, 730000 Gansu China; 8 Key Laboratory of Preclinical Study for New Drugs of Gansu Province, Lanzhou, 730000 Gansu China; 9 School of Basic Medical Sciences of Lanzhou University, School of Medicine, 205 Tianshui Rd South, Lanzhou, 730000 Gansu China

**Retraction Note: Stem Cell Res Ther 10, 60 (2019)**

**https://doi.org/10.1186/s13287-019-1149-5**

The authors have retracted this article. After publication concerns were raised with respect to the data presented in Figure 11B, Figure 11D and Figure 12A. As the authors no longer have access to the original data they have decided to retract this article. All authors agree with this retraction.

